# Aging Will Amplify the Heat-related Mortality Risk under a Changing Climate: Projection for the Elderly in Beijing, China

**DOI:** 10.1038/srep28161

**Published:** 2016-06-20

**Authors:** Tiantian Li, Radley M. Horton, Daniel A. Bader, Maigeng Zhou, Xudong Liang, Jie Ban, Qinghua Sun, Patrick L. Kinney

**Affiliations:** 1 Institute for Environmental Health and Related Product Safety, Chinese Center for Disease Control and Prevention, Beijing, China; 2 Center for Climate Systems Research, Columbia University, New York, USA; 3 The National Center for Chronic and Noncommunicable Disease Control and Prevention, Beijing, China; 4 Institute of Urban Meteorology, China Meteorological Administration (CMA), Beijing, China; 5 Mailman School of Public Health, Columbia University, New York, USA

## Abstract

An aging population could substantially enhance the burden of heat-related health risks in a warming climate because of their higher susceptibility to extreme heat health effects. Here, we project heat-related mortality for adults 65 years and older in Beijing China across 31 downscaled climate models and 2 representative concentration pathways (RCPs) in the 2020s, 2050s, and 2080s. Under a scenario of medium population and RCP8.5, by the 2080s, Beijing is projected to experience 14,401 heat-related deaths per year for elderly individuals, which is a 264.9% increase compared with the 1980s. These impacts could be moderated through adaptation. In the 2080s, even with the 30% and 50% adaptation rate assumed in our study, the increase in heat-related death is approximately 7.4 times and 1.3 times larger than in the 1980s respectively under a scenario of high population and RCP8.5. These findings could assist countries in establishing public health intervention policies for the dual problems of climate change and aging population. Examples could include ensuring facilities with large elderly populations are protected from extreme heat (for example through back-up power supplies and/or passive cooling) and using databases and community networks to ensure the home-bound elderly are safe during extreme heat events.

Global warming and a rapid increase in age in the population are two major global challenges of the 21^st^ century. An increase in global mean surface temperature of 0.3 °C to 4.8 °C has been projected for 2081–2100 relative to 1986–2005^1^. The world population is projected to increase to 9.6–12.3 billion in 2100^2^, with rapid growth in the number and proportion of elderly individuals in both developed and developing countries. Many studies have reported that global warming may result in increasing health risk^3^,^4^,^5^,^6^,^7^,^8^. The health burden of future climate warming could be even higher than climate-focused previous estimates because of the combined impact of higher risks of heat among older adults and a rapidly aging population^9^,^10^. However, the absence of quantitative projections of global warming integrated with demographic changes restricts our understanding of these emerging health risks. There is an urgent need for studies that project the health risk by considering both global warming and population aging, leading to improved understanding of public health intervention policy making, adaption strategy planning, risk communication, and national health plan preparedness focused on elderly populations.

China represents an iconic example of future heat exposure among the elderly due to demographic and climate change. China has the largest population in the world and will be confronted with major challenges related to its aging population in coming decades. According to a report from China’s 2010 census, the population of persons 65 years and above rose to 118.8 million from 100.5 million in 2000^11^. China’s potential support ratio (equal to the number of people aged 20 to 64 divided by the number of people 65 and over) is projected to decline to 1.8 by 2100 from the current level of 7.8^2^. The significant and rapid increase in life expectancy during 1990 to 2013 in different regions of China has occurred during the transition period of economic growth and demographic changes^12^, a period also associated with explosive growth in China’s greenhouse gas emissions.

These demographic changes will interact with increasing heat hazards as well. According to the 3rd National Assessment Report on Climate Change, the annual average temperature of the Chinese mainland area has increased by 0.21–0.25 °C/decade in the past 50–60 years. This warming is projected to accelerate during the 21st century, leading to more frequent extreme heat events^13^, as well as other health impacts associated for example with water shortages and air quality that are beyond the scope of this study^14^.

Beijing is a microcosm of rapid urbanization in China and is representative of the development mode followed by other growing Chinese cities in the future. This city is vulnerable to global warming due to the high concentration of older adults, as well as the urban heat island effect^5^. Beijing is the center of China’s politics, economy, diplomacy, science, technology, and education, all of which are playing a large and growing role in China and the world. Projections for Beijing may have important policy implications for China and analogous cities elsewhere in the world facing the dual problems of climate change and population aging.

A number of prior studies have projected future heat-related mortality in a changing climate^3^,^5^,^8^. However, little work has been done to project the heat-related health risk for older adults, a particularly vulnerable population subgroup^9^,^10^,^15^,^16^. Previous studies have attempted to determine the quantitative projection by considering potential changes in population^17^ and adaptation^3^,^5^,^7^,^18^,^19^,^20^. Very little has been reported on the health risk tradeoffs among heat, population, and adaptation under a changing climate. Few studies have projected heat-related mortality risk by incorporating a full range of climate models/scenarios^8^,^15^. Furthermore, not many heat mortality studies have integrated climate model results from the newly issued representative concentration pathways (RCPs)^4^,^21^,^22^,^23^,^24^,^25^.The objective of our study was to project heat-related mortality for adults 65 years and older in Beijing across 31 statistically downscaled climate models and 2 RCPs in the 2020s, 2050s, and 2080s. By examining a range of scenarios of climate and demographic changes, we aimed to provide a more complete accounting of the magnitude and uncertainties of future heat-related health risk under a changing climate.

## Methods

We first estimated the exposure-response relationship between observed daily mortality of persons 65 years of age and older and daily temperature in Beijing. We obtained downscaled temperature projections from 31 climate models and 2 RCPs, and population projections under three demographic scenarios. These inputs were then combined to estimate future heat-related mortality, which were compared with heat-related mortality in a baseline period.

### Exposure-Response Relationship

#### Data

Historical data of deaths and mean temperature for the years 2008 through 2011 were collected in Beijing. Daily mortality data were obtained from the Chinese Center for Disease Control and Prevention. Daily death counts of persons 65 years of age and older from all internal causes (ICD-10 codes A00-R99) were pooled, excluding accidental causes. Daily Tmean data and other meteorological data were obtained from the China Meteorological Data Sharing Service System for Beijing (station number: 54511), which is the only official meteorological station to represent Beijing meteorological information in the world weather station network. To address potential impacts of air pollution, PM_2.5_ data covering the period between 2008 and 2011 and the O_3_ data covering the period between 2009 and 2011 were obtained from the Beijing Meteorological Bureau, which provides daily PM_2.5_ and O_3_ concentrations from the Beijing Baolian station. Table S1 shows the descriptive statistics for all the variables, and Fig. S1 shows the time series plot of daily mortality of persons 65 years of age and older.

#### Statistical analysis

We used a Poisson regression model combined with a distributed lag non-linear model (DLNM) to examine the impact of temperature on mortality^26^,^27^.We controlled for day of the week as a categorical variable. We controlled for season and long-term trend using a natural cubic spline with 7 df per year for time.

We used a DLNM to examine the non-linear and delayed effects of temperature on mortality because prior studies have shown that temperature can have a lagged effect on mortality^28^,^29^,^30^. Moreover, the relationship between temperature and mortality is non-linear^30^. The DLNM is developed based on “cross-basis” function, which allows simultaneous estimation of the non-linear effects of temperature at each lag and the non-linear effects across lags. The relationship between temperature and mortality can be assessed at each temperature point and lag. The cumulative effect in the delayed contributions can also be calculated^27^.

We modeled the exposure-response curve with a natural cubic spline with 3 internal knots placed at equally spaced values, and the lag-response curve with a natural cubic spline with the 3 internal knots placed at equally spaced values in the log scale. We selected a lag of 14 days to model the effects of temperature on mortality because previous studies have suggested that the effects of temperature on heat usually have lag effects, which are potentially affected by mortality displacement^28^,^30^,^31^,^32^. A minimum mortality risk temperature (MMT) of 21.4 °C was derived from the overall cumulative exposure-response association and used as the reference temperature to calculate relative risks.

Sensitivity analyses for exposure-response relationship investigated potential modifications related to modeling choices, exposure metrics, and air pollution (PM_2.5_ and ozone).

The analyses were performed using R software (version 3.2) and the DLNM package.

### Temperature Projections

Temperature projections were developed by first extracting downscaled outputs from 31 global scale general circulation models (GCMs) used in the Intergovernmental Panel on Climate Change Fifth Assessment report, in conjunction with 2 representative concentration pathways^33^.

The fifth IPCC report introduced a set of new scenarios named RCPs, which are the product of an innovative collaboration between integrated assessment modelers, climate modelers, terrestrial ecosystem modelers, and emission inventory experts^1^,^33^. The following 2 RCPs were selected in this study: RCP4.5 is a stabilization scenario in which the total radiative forcing is stabilized shortly after 2100, and it assumes a lower population growth rate, high income and rapid technology distribution^33^. RCP8.5 is characterized by increasing greenhouse gas emissions over time, which assumes a high population growth rate, lower income, and a lower technology distribution rate^34^.

The climate model outputs from the WCRP CMIP5 multi-model dataset^35^, were further downscaled to 1/2-degree resolution through bias-correction and spatial disaggregation (BCSD). The BCSD projections were obtained online^36^. The spatial average of the BCSD land-based grid boxes corresponding to the latitude and longitude coordinates of Beijing (latitude: 39°26′–41°03′; longitude: 115°25′–117°30′) was used to create 12 (January–December) change factors based on each of the 30-year future periods relative to the same GCMs 30-year base period. Monthly change factors were developed for mean temperatures. These change factors were then applied to the corresponding daily Beijing weather data (mean temperature changes to observed means) to create a future projection. The climate model baseline from 1970–1999 is compared to the same model’s 30 year future period, in order to develop a delta for each calendar month and GCM. This approach assumes inter-annual and high frequency intra-annual temperature variability will remain constant. However, possible changes in the annual temperature cycle are captured^37^.

The resulting 31 temperature projections for daily Tmean from 2010 to 2099 are based on three 30-year time slices, which are 2020s (2010–2039), 2050s (2040–2069) and 2080s (2070–2099), and for a baseline period 1980s (1970–1999). The 31 GCMs are described in Table S2.

### Population

The population of persons 65 and older in the baseline period (1980s) was obtained from the Beijing Municipal Bureau of Statistics^38^. Two population projection scenarios were used to estimate heat-related mortality in the 2020s, 2050s, and 2080s. In the “no change” scenario, the population and composition of the population were both held constant at baseline levels throughout the projection period. For the “aging” scenario, we used the low, medium, and high variant scenarios of population growth among persons 65 year and older for China developed by the United Nations (UN)^39^. Under the low variant scenario, fertility is projected to remain 0.5 children below the fertility in the medium variant over most of the projection period. Under the high variant, fertility is projected to remain 0.5 children above the fertility in the medium variant over most of the projection period. Under the medium variant, total fertility in all countries is assumed to converge eventually toward a level of 1.85 children per woman^39^. Baseline mortality rates, which excluded deaths attributable to external causes, were obtained from the Chinese Center for Diseases Control and Prevention. We held baseline mortality rates constant in our projection.

### Adaptation

Future heat adaptation was also applied in this study. Recent evidence suggests that the heat-mortality relationship may be diminishing over time due to adaptation phenomena^3^,^5^,^9^,^15^,^40^,^41^. Previous studies have shown that, over time or in warmer climates, the temperature-mortality curves maintained a similar shape but with the heat slope decreasing, while the MMT became higher^42^. A recent paper showed that in New York City, the heat effect on persons 65 years and older decreased by approximately 30% in the 20th century^9^. On the grounds that New York City had transitioned dramatically over this long period from no air conditioning to approaching (though certainly not meeting) full saturation of air conditioning, for scenario purposes we set this same 30% as one scenario of heat effect reductions for the comparable time period covered by our projections. For testing the higher level of adaptation, we set 50% adaptation rate as the highest adaptation scenario. In this study, we set 0%, 5%, 15%, 30% and 50% reduction of the heat effect as 5 adaptation scenarios. Therefore, we shifted the temperature-mortality relationship by reducing 5%, 15%, 30% and 50% of the heat effect, and moved MMT simultaneously for modeling the adapted temperature-mortality relationship.

### Mortality Projection

Projected mortality impacts were estimated using modeled daily Tmean. For any day with Tmean greater than MMT, the change in mortality was calculated relative to MMT for the heat effect. Daily additional heat-related deaths were computed as:


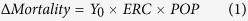


Where Δ*Mortality* is daily temperature-related additional deaths, Y_0_ is baseline daily mortality rate (per 100,000 population), and POP is population. ERC is attributable percentage change in mortality for a specified change in temperature, derived from the calculated relative risk at each temperature from the statistical analysis of observed data as described above.

We computed heat-related daily deaths in this way for each time period (1980s, 2020s, 2050s, and 2080s), and then computed the average number of heat-related deaths per year. We also computed percent changes in annual average deaths from the 1980s to future time periods.

### Uncertainty Analyses

We used the ANOVA-type estimation of variance components (VC) method in the uncertainty analysis. We performed the analysis in different period respectively for understanding the influence of different factor on heat-related mortality projection. These factors include the 2 RCPs, 31 GCMs, 4 population scenarios and 5 adaptation scenarios.

The analyses were conducted using R software (version 3.2) and the VCA package.

## Results

**Figure 1:**
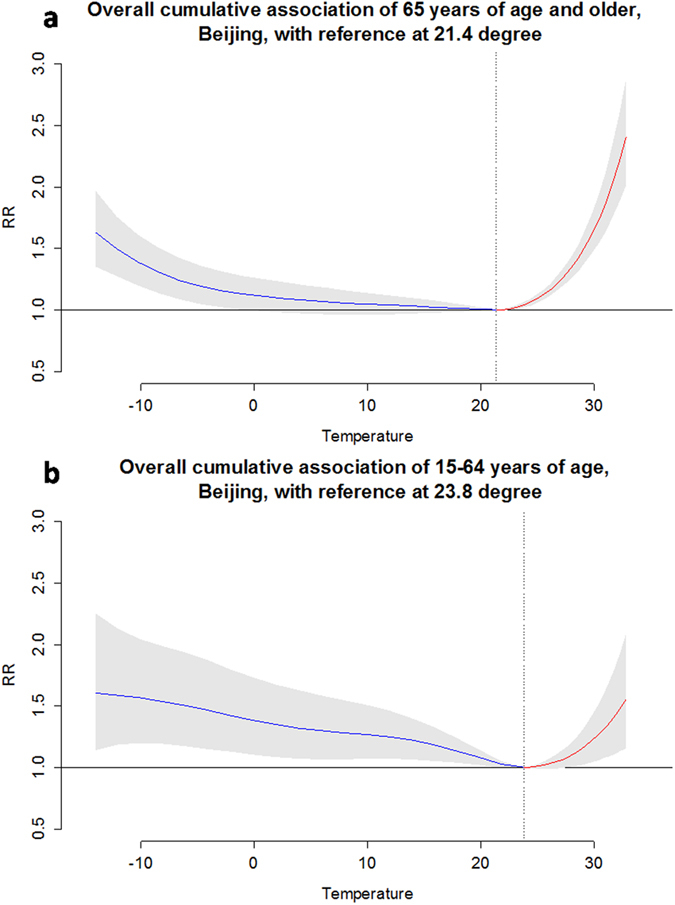
Exposure-response curves for temperature-related mortality of persons 65 years of age and older, and for all other persons.

Figure 1 shows the overall cumulative exposure-response curve between daily mean temperature and the mortality of persons 65 years of age and older derived using the non-linear distributed lag models. The curves are U-shaped at lag 0–14 with the MMT at 21.4 °C. For comparison, we also show the corresponding curve for persons age 15–64. Temperatures above the MMT were associated with excess mortality. We linked the exposure-response function for temperatures above the MMT to the 31 modeled projections of daily temperatures to estimate the numbers of heat-related deaths in both the baseline and future time periods. Results from the sensitivity analysis of dose-response relationship are presented in Tables S3, S4, S5 and S6. Results were not affected substantially in sensitivity models. We examined different cumulative lags, consistent with recent studies^43^. The effect estimates of other lag structures (0–7 days, 0–21 days, and 0–28 days) were not dissimilar to that at lag 0–14 (Supplementary Table S3).

**Figure 2:**
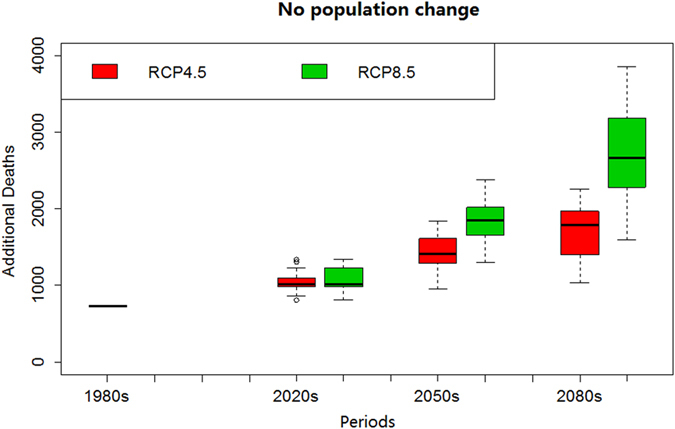
Distribution of heat-related annual deaths in the 1980s, 2020s, 2050s, and 2080s for 31 climate models and the RCP4.5 and RCP8.5 scenarios, with no change over time in population.

Figure 2 graphically summarizes the projected heat-related deaths from the 1980s to 2080s with the population held constant at the 1980s level (no population change and no adaptation scenario). Here, heat-related deaths increase from the 1980s to 2080s, more rapidly so for the RCP8.5 compared with the RCP4.5 climate change scenario. Except for the 2020s, the projected deaths are significantly different (p < 0.01) between RCP4.5 and RCP8.5 in the 2050s and 2080s. Moreover, the projected deaths are significantly different (p < 0.01) among 2020s, 2050s, and 2080s within each RCP. Under the RCP8.5 scenario, heat-related deaths are projected to increase by a median 39.1% (11.1% to 83.3%) in the 2020s and 264.9% (117.5% to 427.3%) in the 2080s, compared with the 1980s.

**Figure 3:**
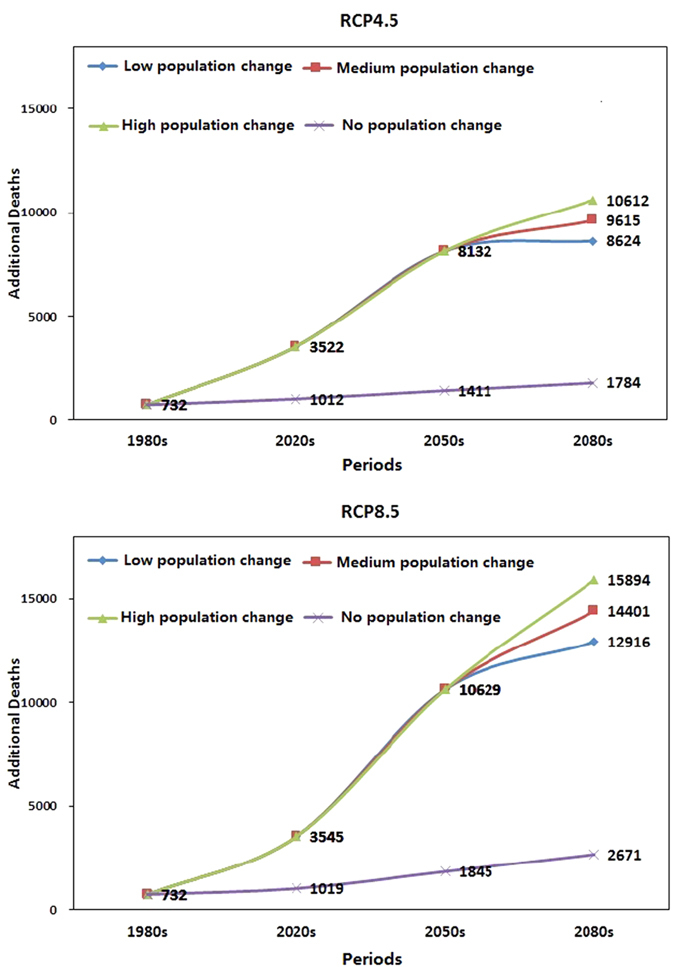
Projection of heat-related deaths (median of 31 models) in the 1980s, 2020s, 2050s, and 2080s for different population variant scenarios and the RCP4.5 and RCP8.5 scenarios.

Projected annual heat-related deaths taking into account projected growth in the population of persons 65 years and older are shown in Fig. 3, along with the results assuming no population change. The median deaths estimated across the 31 climate models are plotted. Boxplots of the distribution of estimates across models are presented in Fig. S2. Under the medium UN population scenario, heat-related deaths increase rapidly from the 1980s to 2050s under both the RCP4.5 and RCP8.5 scenarios, and the impacts are far greater than under the no change population scenario. Projected impacts increased on median by 13.5 (9.2 to 17.4) times of annual heat-related deaths in the 2050s and RCP8.5 with the population median variation compared with the 1980s with the population no variation. In the 2080s, the median heat-related deaths increased further compared with the 2050s, by 18.2% and 35.5% for the RCP4.5 and RCP 8.5 scenarios, respectively. Similar patterns were observed for the distribution of projections (Fig. S2). In the absence of demographic changes in the population, we projected 2671 (1592 to 3860) heat-related deaths per year for persons 65 or older, which showed a 264.9% (117.5% to 427.3%) increase of heat-related mortality in this age group in the 2080s compared with the 1980s under the RCP8.5 scenario. Considering the fact that the future population is growing (under the medium population variation scenario), the projection is on median 5.4 times (14,401 heat-related deaths per year) the previous estimate for the 2080s and RCP8.5 scenario (without considering the demographic changes), or approximately an1867% (1072% to 2743%) increase compared with the baseline period.

**Figure 4:**
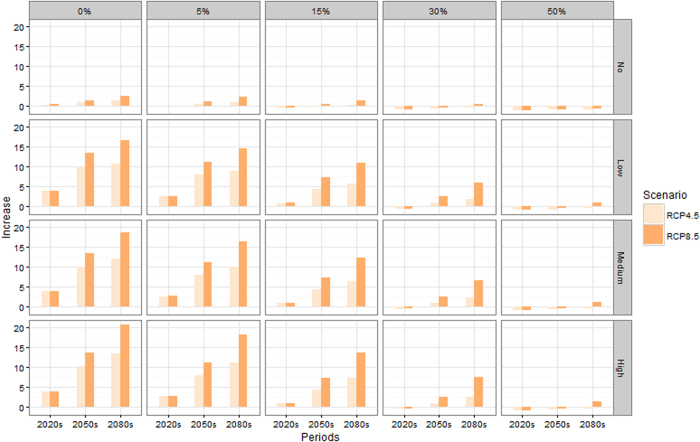
Times increases from 1980s (median of 31 models) of heat-related deaths in the 2020s, 2050s, and 2080s for different population variant scenarios and the RCP4.5 and RCP8.5 scenarios with different adaptation scenarios. (Rows indicate the population scenarios; Columns indicate the adaptation scenarios).

Projections with different adaptations to heat are shown in Fig. 4 (projected number of deaths is shown in Fig. S3). In the 2080s and 2050s with both RCP8.5 and RCP4.5, under the three UN population variations, the heat-related deaths increased under 3 adaptation scenarios (adaption to heat increasing about 5%, 15%, and 30%, respectively) compared with the 1980s. However, with the scenario of adaptation to heat increasing 50%, under the three UN population variations, the heat-related death increased only in the 2080s with RCP8.5. In the 2020s with both the RCP8.5 and RCP4.5 scenarios, the heat-related deaths increased under the 5% and 15% adaptation scenarios, but decreased under the 30% and 50% adaptation scenario when compared with the 1980s. The largest increase in heat-related death is more than 20-fold compared with the 1980s under the no adaptation to heat and population high variation scenario in the 2080s and RCP8.5. In the 2080s, even with the 30% and 50% adaptation rate assumed in our study, the increase in heat-related death is approximately 7.4 times and 1.3 times larger than in the 1980s respectively under a scenario of high population and RCP8.5. Results from the uncertainty analysis in different periods based on the variance composition method are presented in Fig. S4. In all 3 periods, the 5 adaptation scenarios were attributed the highest uncertainty, followed by the 4 population scenarios. The uncertainty attributed to 2 RCPs and 31 GCMs increased through time.

## Discussion

This study is the first to project the health effect of rising temperatures under a changing climate on the fast-aging population in the world’s most populous country over the 21st century. Previous works have projected increases in heat-related mortality under the changing climate in Europe and the U.S., but seldom with age-specific population projections^5^. Our results in Beijing showed similar increasing trends to those found in previous works, while the significant differences in the absolute numbers of the projected deaths were due to the fact that China has the largest population in the world. We found that ignoring adaptation and demographic changes among older adults who are most susceptible to heat leads to substantial differences in estimates of future heat-related mortality. This study provides the first insight into the important synergy between an aging population and global warming in China.

Our exposure response function was qualitatively similar to previous studies. The minimum mortality occurred at approximately the 70^th^ percentile across the range of temperatures, consistent with previous studies, which reported MMTs in the 60–80^th^ percentile range^4^,^43^,^44^,^45^,^46^. As previously mentioned, heat effects were strongest at short time lags, whereas cold effects extended over multiple lag days.

Nearly all previous studies assumed a constant population to experience the health risk^3^,^4^,^8^,^15^. Four studies in the United States, Europe, and Australia considered the simple demographic trend of total population in the future rather than using scenarios with the age-specific population changes for a long period in the projection. Most of them projected only to the middle of the century and did not compare across alternative population scenarios^5^,^6^. Our study introduced the newly issued UN demographic scenarios to project heat-related mortality over almost 100 years. These studies also did not show the differences in projection results between the models assuming constant population and those assuming demographic changes. For countries like China with the challenges of large population increases and significant aging in the future, the scenario with population changes should be incorporated in the projection process. We found that in high population variation, heat-related mortality would be approximately 2.5 times higher by the 2020s, 4.8 times higher by the 2050s, and 5 times higher by the 2080s compared with a scenario of no population change. Our finding for the first time provides quantitative evidence comparing projections with and without demographic changes in China. Such more realistic heat-related mortality projections in the context of an aging population in China may help foster adaptation policies to protect the elderly from heat, such as warning systems and public health campaigns focused on the elderly. Furthermore, our findings and methods could provide a reference for other countries experiencing rapid population growth and demographic transitions.

The temperature-mortality relationship may not remain stable over time because of adaptation to heat and other factors. For example, the heat effect in New York City declined dramatically over the 20^th^ century, especially for individuals 65 years of age and older^9^. Also it has been demonstrated that the mortality risk due to heat appeared to decrease over time in several countries^41^. However, modeling future adaptation remains a significant challenge. Most prior studies assume no adaptation over time^4^,^6^,^8^. Some studies examined adaptation by using the exposure-response relationship from analogue cities or analogue summers^3^,^18^,^19^. Another approach is to shift the current temperature-mortality relationships to the future by increasing the temperature of MMT, while leaving the slope of the temperature-mortality curve unchanged^7^,^47^. A recent study revealed that heat adaptation not only manifests as MMT increase, but also displays relative risk reduction^9^. We modeled adaptation by shifting both the MMT and shape of the temperature-mortality curve to derive 4 adaptation scenarios. Compared with the no adaptation scenario, the heat-related mortality decreased under the different adaptation scenarios. A 30% adaptation scenario chosen in this study has been observed historically in the United States during the 20th century. A 30% level of adaptation would not completely offset the projected heat impact from climate change on the elderly in Beijing. A higher level of adaptation scenario (50% adaptation) was also explored in this study, which tended to more fully offset the projected heat impact from climate change on the elderly in Beijing even under the RCP 8.5 and high population scenario. Future studies could explore additional plausible upper bounds for the adaptation effect, in Beijing and elsewhere.

Climate change is another key uncertainty in heat-related mortality projections^5^. However, only recently have studies begun to take advantage of the large number of GCMs now available^8^. We applied downscaled outputs from 31 different GCMs and 2 future RCPs. Research is transitioning from the SRES greenhouse gas concentration scenarios to the RCPs^4^,^21^,^22^,^23^,^24^,^25^, which have certain methodological advantages. By using RCP 4.5 and 8.5, we are able to sample a broader range of possible outcomes than was possible in SRES (since RCP8.5 was run with more climate models than the most comparable high end SRES scenario (A1FI)); at the lower end, RCP4.5 is generally comparable to the SRES B1 scenario. The median projection from the RCP4.5 and the increasing RCP8.5 scenarios showed growing divergence with time. While it is impossible to sample the full range of possible climate outcomes, our results from a wide range of models and multiple RCP scenarios provide important uncertainty information for adaptation policy planning. Future work could explore the health implications of extreme temperature scenarios consistent with the controversial idea that changing atmospheric dynamics^48^ could lead to faster increases in extreme heat than are projected by GCMs under the RCPs^49^.

While we incorporated a rich range of uncertainties related to climate change, population demographics and adaptation, our work has some limitations. We assumed a constant population mortality rate in our projection. Changes in general health, access to health care, socio-economic status, and public health education and prevention are additional important uncertainties^5^,^15^. Addressing adaptation uncertainty is an urgent and complex problem in the field of heat-related projection^15^. Additional methods for modeling adaptations are needed to reduce uncertainty in heat-related projections and to recursively identify and implement key actions that protect public health. These actions may include adaptive measures to help the elderly cope with heat, such as reducing isolation and enhancing access to cooling resources, as well as mitigation of heat in cities through built environment modifications and large-scale climate policy.

An aging population will substantially enhance the burden of heat-related health risks in a warming climate in China. This study provides the first evidence of an important hybrid question of the aging population and global warming in this century. These findings strongly support the need for countries in making public health intervention policy for the dual problems of climate change and aging population. A range of models and multiple RCPs here enable a future health risk projection with articulation of key uncertainty, which could provide more credible information support in implementing policies that mitigate climate change and promote public health. In addition, future research may be needed to consider the effects of rapid urbanization of the city, which may be on the same order of magnitude as effects from global-scale greenhouse gas-forced climate change^50^, and could also lead to increases in future heat-related deaths completely independent of any global-scale processes^51^. Finally, narrower data subsets of heat-specific health outcomes should be explored rather than the use of all causes mortality records in future research.

## Additional Information

**How to cite this article**: Li, T. *et al.* Aging Will Amplify the Heat-related Mortality Risk under a Changing Climate: Projection for the Elderly in Beijing, China. *Sci. Rep.* **6**, 28161; doi: 10.1038/srep28161 (2016).

## Supplementary Material

Supplementary Information

## References

[b1] Intergovernmental Panel on Climate Change. Climate Change 2013: The Physical Science Basis. United Kingdom and New York, NY, USA: Cambridge University Press (2013).

[b2] Gerland P. *et al.* World population stabilization unlikely this century. Science 346, 234–237 (2014). 25301627 10.1126/science.1257469 PMC4230924

[b3] Knowlton K. *et al.* Projecting Heat-Related Mortality Impacts Under a Changing Climate in the New York City Region. Am. J. Public Health 97, 2028–2034 (2007). 17901433 10.2105/AJPH.2006.102947 PMC2040370

[b4] Petkova E. P., Horton R. M., Bader D. A. & Kinney P. L. Projected heat-related mortality in the U.S. urban northeast. Int. J. Environ. Res. Public Health 10, 6734–6747 (2013). 24300074 10.3390/ijerph10126734 PMC3881138

[b5] Huang C. *et al.* Projecting Future Heat-Related Mortality under Climate Change Scenarios: A Systematic Review. Environ. Health Perspect. 119, 1681–1690 (2011). 21816703 10.1289/ehp.1103456 PMC3261978

[b6] Hajat S., Vardoulakis S., Heaviside C. & Eggen B. Climate change effects on human health: projections of temperature-related mortality for the UK during the 2020s, 2050s and 2080s. J. Epidemiol. Community Health 68, 641–648 (2014). 24493740 10.1136/jech-2013-202449

[b7] Gosling S., McGregor G. & Lowe J. Climate change and heat-related mortality in six cities Part 2: climate model evaluation and projected impacts from changes in the mean and variability of temperature with climate change. Int. J. Biometeorol. 53, 31–51 (2008). 19052780 10.1007/s00484-008-0189-9

[b8] Li T., Horton R. & Kinney P. Projections of seasonal patterns in temperature- related deaths for Manhattan, New York. Nat. Clim. Chang. 3, 717–721 (2013). 24910717 10.1038/nclimate1902 PMC4045618

[b9] Petkova E. P., Gasparrini A. & Kinney P. L. Heat and mortality in New York City since the beginning of the 20th century. Epidemiology 25, 554–560 (2014). 24802366 10.1097/EDE.0000000000000123 PMC4096340

[b10] Gronlund C. J., Zanobetti A., Schwartz J. D., Wellenius G. A. & O’Neill M. S. Heat, heat waves, and hospital admissions among the elderly in the United States, 1992–2006. Environ. Health Perspect. 122, 1187–1192 (2014). 24905551 10.1289/ehp.1206132 PMC4216145

[b11] Peng X. China’s demographic history and future challenges. Science 333, 581–587 (2011). 21798939 10.1126/science.1209396

[b12] Zhou M. G. *et al.* Cause-specific mortality for 240 causes in China during 1990–2013: a systematic subnational analysis for the Global Burden of Disease Study 2013. Lancet 387, 251–272 (2016). 26510778 10.1016/S0140-6736(15)00551-6

[b13] Collins M. *et al.* Climate Change 2013: The Physical Science Basis. Contribution of Working Group I to the Fifth Assessment Report of the Intergovernmental Panel on Climate Change (eds Stocker T. F. *et al.*) Ch.12 Long-term Climate Change: Projections, Commitments and Irreversibility, (Cambridge University Press 2013).

[b14] Hijioka Y. *et al.* Climate Change 2014: Impacts, Adaptation, and Vulnerability. Part B: Regional Aspects. Contribution of Working Group II to the Fifth Assessment Report of the Intergovernmental Panel on Climate Change (eds Barros V. R. *et al.*) Ch. Asia, 1327–1370 (Cambridge University Press 2014).

[b15] Kinney P., O’Neill M., Bell M. & Schwartz J. Approaches for estimating effects of climate change on heat-related deaths: challenges and opportunities. Environ. Sci. Policy 11, 87–96 (2008).

[b16] Bobb J. F., Obermeyer Z., Wang Y. & Dominici F. Cause-specific risk of hospital admission related to extreme heat in older adults. JAMA 312, 2659–2667 (2014). 25536257 10.1001/jama.2014.15715 PMC4319792

[b17] Sheridan S. C., Allen M. J., Lee C. C. & Kalkstein L. S. Future heat vulnerability in California, Part II: projecting future heat-related mortality. Climatic chang. 115, 311–326 (2012).

[b18] Hayhoe K. *et al.* Emissions pathways, climate change, and impacts on California. P. Natl. Acad. Sci. USA 101, 12422–12427 (2004). 10.1073/pnas.0404500101 PMC514653 15314227

[b19] Cheng C. *et al.* Differential and combined impacts of extreme temperatures and air pollution on human mortality in south–central Canada. Part II: future estimates. Air Qual. Atmos. Health 1, 223–235 (2009).

[b20] Hondula D. M., Balling R. C. Jr., Vanos J. K. & Georgescu M. Rising temperatures, human health, and the role of adaptation. Current Clim. Chang. Rep. 1, 144–154 (2015).

[b21] Kingsley S. L., Eliot M. N., Gold J., Vanderslice R. R. & Wellenius G. A. Current and projected heat-related morbidity and mortality in Rhode Island. Environ. Health Perspect. 124, 460–467 (2016). 26251954 10.1289/ehp.1408826 PMC4829994

[b22] Oleson K. W., Anderson G. B., Jones B., McGinnis S. A. & Sanderson B. Avoided climate impacts of urban and rural heat and cold waves over the US using large climate model ensembles for RCP8.5 and RCP4.5. Climatic Chang. doi: 10.1007/s10584-015-1504-1 (2015). PMC5839517 29520121

[b23] Wu J. *et al.* Estimation and uncertainty analysis of impacts of future heat waves on mortality in the eastern United States. Environ Health Perspect. 122, 10–16 (2014). 24192064 10.1289/ehp.1306670 PMC3888568

[b24] Kim D. W., Deo R. C., Chung J. H. & Lee J. S. Projection of heat wave mortality related to climate change in Korea. *Nat*. Hazards 80, 1–15 (2016).

[b25] Kim Y., Kim S. & Liu Y. The impact of climate change on heat-related mortality in six major cities, South Korea, under representative concentration pathways (RCPs). Front. Environ. Sci. 2, 1–11 (2014). 10.3389/fenvs.2014.00003 PMC8204571 34136496

[b26] Armstrong B. Models for the relationship between ambient temperature and daily mortality. Epidemiology 17, 624–631 (2006). 17028505 10.1097/01.ede.0000239732.50999.8f

[b27] Gasparrini A., Armstrong B. & Kenward M. G. Distributed lag non-linear models. Stat. Med. 29, 2224–2234 (2010). 20812303 10.1002/sim.3940 PMC2998707

[b28] Braga A. L., Zanobetti A. & Schwartz J. The effect of weather on respiratory and cardiovascular deaths in 12 U.S. cities. Environ. Health Perspect. 110, 859–863 (2002). 12204818 10.1289/ehp.02110859 PMC1240983

[b29] Zanobetti A., Wand M., Schwartz J. & Ryan L. Generalized additive distributed lag models: quantifying mortality displacement. Biostatistics 3, 279–292 (2000). 12933509 10.1093/biostatistics/1.3.279

[b30] Guo Y., Barnett A. G., Pan X., Yu W. & Tong S. The impact of temperature on mortality in Tianjin, China: a case-crossover design with a distributed lag nonlinear model. Environ. Health Perspect. 119, 1719–1725 (2011). 21827978 10.1289/ehp.1103598 PMC3261984

[b31] Tong S., Ren C. & Becker N. Excess deaths during the 2004 heatwave in Brisbane, Australia. Int. J. Biometeorol. 54, 393–400 (2010). 20049484 10.1007/s00484-009-0290-8

[b32] Goldberg M. S., Gasparrini A., Armstrong B. & Valois M.-F. F. The short-term influence of temperature on daily mortality in the temperate climate of Montreal, Canada. Environ. Res. 111, 853–860 (2011). 21684539 10.1016/j.envres.2011.05.022

[b33] vanVuuren V. D., Edmonds J., Kainuma M. & Riahi K. The representative concentration pathways: an overview. Climatic Chang. 109, 5–31 (2011).

[b34] Moss R. *et al.* Towards new scenarios for analysis of emissions, climate change, impacts, and response strategies. Conserv. Lett. 5, 399–406 (2012).

[b35] Taylor K. E., Stouffer R. J. & Meehl G. A. An overview of CMIP5 and the experiment design. B. Am. Meteorol. Soc. 93, 485–498 (2012).

[b36] Reclamation. Downscaled CMIP3 and CMIP5 Climate and Hydrology Projections: Release of Downscaled CMIP5 Climate Projections, Comparison with preceding Information, and Summary of User Needs, prepared by the U.S. Department of the Interior, Bureau of Reclamation, Technical Services Center, Denver, Colorado. 47p (2013).

[b37] Ballester J., Giorgi F. & Rodó X. Changes in European temperature extremes can be predicted from changes in PDF central statistics. Climatic Chang. 98, 277–284 (2009).

[b38] The Beijing Municipality Statistical Bureau. Beijing Statistical Yearbook. Beijing (2013).

[b39] United Nations, Department of Economic and Social Affairs. World Population Prospects: The 2012 Revision, Methodology of the United Nations Population Estimates and Projections. New York (2014).

[b40] Davis R. E., Knappenberger P. C., Michaels P. J. & Novicoff W. M. Changing heat-related mortality in the United States. Environ. Health Perspect. 111, 1712–1718 (2003). 14594620 10.1289/ehp.6336 PMC1241712

[b41] Gasparrini A. *et al.* Temporal Variation in Heat-Mortality Associations: A Multicountry Study. Environ. Health Perspect. 123, 1200–1207 (2015). 25933359 10.1289/ehp.1409070 PMC4629745

[b42] Hajat S. & Kosatky T. Heat-related mortality: a review and exploration of heterogeneity. J. Epidemiol. Commun. Health 64, 753–760 (2010). 10.1136/jech.2009.087999 19692725

[b43] Guo Y. *et al.* Global variation in the effects of ambient temperature on mortality: a systematic evaluation. Epidemiology 25, 781–789 (2014). 25166878 10.1097/EDE.0000000000000165 PMC4180721

[b44] Guo Y., Punnasiri K. & Tong S. Effects of temperature on mortality in Chiang Mai city, Thailand: a time series study. Environ. Health 11, 36–44 (2012). 22613086 10.1186/1476-069X-11-36 PMC3391976

[b45] Honda Y. *et al.* Heat-related mortality risk model for climate change impact projection. Environ. Health Prev. Med. 19, 56–63 (2014). 23928946 10.1007/s12199-013-0354-6 PMC3890078

[b46] Chen R. *et al.* Both low and high temperature may increase the risk of stroke mortality. Neurology 81, 1064–1070 (2013). 23946311 10.1212/WNL.0b013e3182a4a43c PMC3795588

[b47] Dessai S. Heat stress and mortality in Lisbon Part II. An assessment of the potential impacts of climate change. Int. J. Biometeorol. 48, 37–44 (2003). 12750971 10.1007/s00484-003-0180-4

[b48] Petoukhov V., Rahmstorf S., Petri S. & Schellnhuber H. J. Quasiresonant amplification of planetary waves and recent Northern Hemisphere weather extremes. P. Natl. Acad. Sci. USA 110, 5336–5341 (2013). 10.1073/pnas.1222000110 PMC3619331 23457264

[b49] Coumou D., Lehmann J. & Beckmann J. The weakening summer circulation in the Northern Hemisphere mid-latitudes. Science 348, 324–327 (2015). 25765067 10.1126/science.1261768

[b50] Georgescu M., Morefield P. E., Bierwagen B. G. & Weave C. P. Urban adaptation can roll back warming of emerging megapolitan regions. P. Natl. Acad. Sci. USA 111, 2909–2914 (2014). 10.1073/pnas.1322280111 PMC3939866 24516126

[b51] Hondula D. M., Georgescu M. & Balling R. C. Challenges associated with projecting urbanization-induced heat-related mortality. Sci. Total Environ. 490, 538–544 (2014). 24880543 10.1016/j.scitotenv.2014.04.130

