# Supplementary material for: Aging Will Amplify the Heat-related Mortality Risk under a Changing Climate: Projection for the Elderly in Beijing, China
Source: Sci Rep. 2016 Jun 20;6:28161. doi: 10.1038/srep28161 (PMC4913346; doi:10.1038/srep28161)
Supplement: Supplementary Information [file srep28161-s1.pdf]

Supplementary material for the article:

Aging Will Amplify the Heat-related Mortality Risk under a Changing Climate: Projection for the Elderly in Beijing, China

Tiantian Li[1], Radley M. Horton[2], Daniel A. Bader[2], Maigeng Zhou[3], Xudong Liang[4], Jie Ban[1], Qinghua Sun[1], Patrick L. Kinney[5]

[1]Institute for Environmental Health and Related Product Safety, Chinese Center for Disease Control and Prevention, Beijing, China

[2]Center for Climate Systems Research, Columbia University, New York, USA

[3]The National Center for Chronic and Noncommunicable Disease Control and Prevention, Beijing, China

[4]Institute of Urban Meteorology, China Meteorological Administration (CMA), Beijing

[5]Mailman School of Public Health, Columbia University, New York, USA

***Corresponding Authors:**

Tiantian Li, Institute for Environmental Health and Related Product Safety, Chinese Center for Disease Control and Prevention, No. 7 Panjiayuan Nanli, Chaoyang District, Beijing, 100021 China. Telephone: 00861050930211. Email: tiantianli@gmail.com;

Patrick L. Kinney, Mailman School of Public Health, Columbia University, New York, USA, 722 West 168th Street, Room 1104E, New York, NY 10032 USA. Telephone: 0012123053663. Email: plk3@cumc.columbia.edu

Supplementary Tables

Table S1. Mean, range, and specific percentiles for daily variables

	N	Mean	Min	25th	75th	Max
Daily Mean Temperature (℃)	1461	12.4	-14.0	1.6	23.1	32.8
Daily Max Temperature (℃)	1461	18.4	-8.9	8.0	29.0	41.0
Daily Min Temperature (℃)	1461	6.6	-19.0	-4.0	17.9	26.7
Daily Death of persons 65 years of age and older (ICD-10 Code A00-R99)	1461	145	86	127	161	221
Daily $PM_{2.5}$ Concentration (μg/m^3)	1333	74.8	10.4	42.9	96.6	294.2
Daily O_3 Concentration (μg/m^3)	1035	20.7	0.1	6.4	30.7	84.6

Table S2. Global climate models used in this study

**Climate Model Acronym**	**Institution**	**Atmospheric Resolution (latitude × longitude)**
ACCESS1-0	CSIRO (Commonwealth Scientific and Industrial Research Organization, Australia), and BOM (Bureau of Meteorology, Australia)	1.25 °×1.875 °
BCC-CSM1-1	Beijing Climate Center, China Meteorological Administration	2.8125 °×2.8125 °
BCC-CSM1-1-M	Beijing Climate Center, China Meteorological Administration	T106
BNU-ESM	College of Global Change and Earth System Science, Beijing Normal University	2.8125 °×2.8125 °
CanESM2	Canadian Centre for Climate Modelling and Analysis	1.875 °×1.875 °
CCSM4	National Center for Atmospheric Research	0.9 °×1.25 °
CESM1-BGC	National Science Foundation, Department of Energy, National Center for Atmospheric Research	0.9 °×1.25 °
CESM1-CAM5	National Science Foundation, Department of Energy, National Center for Atmospheric Research	0.9 °×1.25 °
CMCC-CM	Centro Euro-Mediterraneo per I Cambiamenti Climatici	0.75 °×0.75 °
CNRM-CM5	Centre National de Recherches Meteorologiques / Centre Europeen de Recherche et Formation Avancees en Calcul Scientifique	2.8 °×2.8 °
CSIRO-Mk3-6-0	Commonwealth Scientific and Industrial Research Organisation in collaboration with the Queensland Climate Change Centre of Excellence	1.875 °×1.875 °
FGOALS-G2	LASG, Institute of Atmospheric Physics, Chinese Academy of Sciences; and CESS, Tsinghua University	2.8125 °×2.8125 °
FIO-ESM	The First Institute of Oceanography, SOA, China	2.8125 °×2.8125 °
GFDL-CM3	Geophysical Fluid Dynamics Laboratory	200 km×200 km

GFDL-ESM2G	Geophysical Fluid Dynamics Laboratory	2°×2.5°
GFDL-ESM2M	Geophysical Fluid Dynamics Laboratory	2°×2.5°
GISS-E2-R	NASA Goddard Institute for Space Studies	2°×2.5°
HadGEM2-AO	National Institute of Meteorological Research/Korea Meteorological Administration	1.25 °×1.875 °
HadGEM2-CC	Met Office Hadley Centre (additional HadGEM2-ES realizations contributed by Instituto Nacional de Pesquisas Espaciais)	1.25 °×1.875 °
HasGEM2-ES	Met Office Hadley Centre (additional HadGEM2-ES realizations contributed by Instituto Nacional de Pesquisas Espaciais)	1.25 °×1.875 °
INMCM4	Institute for Numerical Mathematics	1.5 °×2 °
IPSL-CM5A-LR	Institut Pierre-Simon Laplace	3.75 °×1.9 °
IPSL-CM5A-MR	Institut Pierre-Simon Laplace	2.5 °×1.25 °
IPSL-CM5B-LR	Institut Pierre-Simon Laplace	3.75 °×1.9 °
MIRCO-ESM	Japan Agency for Marine-Earth Science and Technology, Atmosphere and Ocean Research Institute (The University of Tokyo), and National Institute for Environmental Studies	2.8125 °×2.8125 °
MIROC-ESM-CHEM	Japan Agency for Marine-Earth Science and Technology, Atmosphere and Ocean Research Institute (The University of Tokyo), and National Institute for Environmental Studies	2.8125 °×2.8125 °
MIROC5	Atmosphere and Ocean Research Institute (The University of Tokyo), National Institute for Environmental Studies, and Japan Agency for Marine-Earth Science and Technology	1.40625 °×1.40625 °
MPI-ESM-LR	Max Planck Institute for Meteorology (MPI-M)	1.8 °×1.8 °
MPI-ESM-MR	Max Planck Institute for Meteorology (MPI-M)	1.8 °×1.8 °
MRI-CGCM3	Meteorological Research Institute	320×160
NorESM1-M	Norwegian Climate Centre	1.9 °×2.5 °

Table S3. Sensitivity of heat effects by modeling choices (using the dataset of 2008-2011)

Sensitivity analyses investigated potential confounding by modeling choices. To quantify the cumulative heat effects, we calculated the change in mortality risk comparing the 99th percentile of the temperature distribution to MMT

	**Heat Effect**
Main model	1.62 (95%CI:1.46 -1.79)
Cubic B-spline for exposure-response	1.62 (95%CI:1.46-1.79)
Df for lag-response: 6	1.62 (95%CI:1.46- 1.79)
Lag period: 7 days	1.48 (95%CI:1.38-1.59)
Lag period: 21days	1.41 (95%CI:1.24- 1.61)
Lag period: 28 days	1.35 (95%CI:1.16- 1.58)
Df/year for seasonal control: 6	1.48 (95%CI:1.36-1.62)
Df/year for seasonal control: 10	1.65 (95%CI:1.48-1.84)

Table S4. Sensitivity of heat effects by exposure metrics (using the dataset of 2008-2011)

Sensitivity analyses investigated potential confounding by exposure metrics. To quantify the cumulative heat effects, we calculated the change in mortality risk comparing the 99th percentile of the temperature distribution to MMT

	Heat Effect
Main model	1.62 (95%CI:1.46 -1.79)
Maximum temperature	1.38 (95%CI:1.22 -1.57)
Minimum temperature	1.53 (95%CI:1.32 -1.77)
Heat index	1.57 (95%CI:1.38 -1.79)

Table S5. Sensitivity of Heat Effects by $PM_{2.5}$ (using the dataset of 2008-2011)

Sensitivity analyses investigated potential confounding by $PM_{2.5}$. To quantify the cumulative heat effects, we calculated the change in mortality risk comparing the 99th percentile of the temperature distribution to MMT

	**Heat Effect**
Main model	1.62 (95%CI:1.46 -1.79)
With $PM_{2.5}$	1.63 (95%CI:1.47- 1.80)

Table S6. Sensitivity of Heat Effects by O_3 (using the dataset of 2009-2011)

Sensitivity analyses investigated potential confounding by O_3. To quantify the cumulative heat effects, we calculated the change in mortality risk comparing the 99th percentile of the temperature distribution to MMT

	**Heat Effect**
Main model	1.66 (95%CI: 1.47- 1.87)
With O_3	1.78 (95%CI: 1.56-2.04)

Supplementary Figures

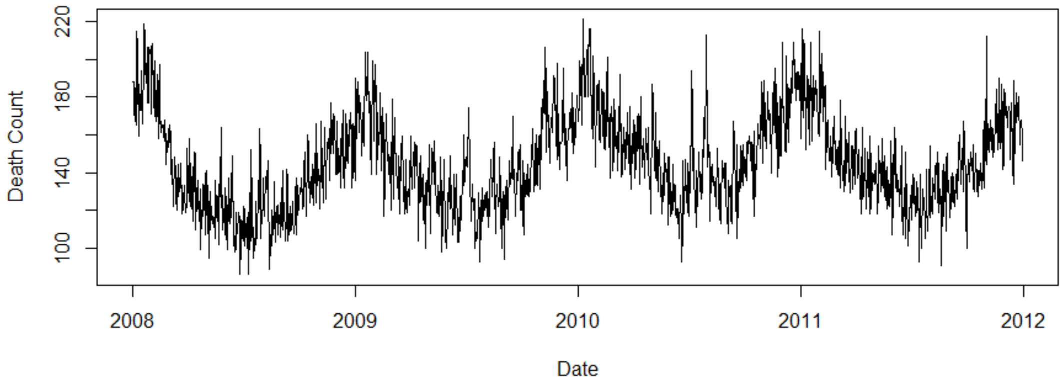

Figure S1. Time series plot of daily mortality of persons 65 years of age and older

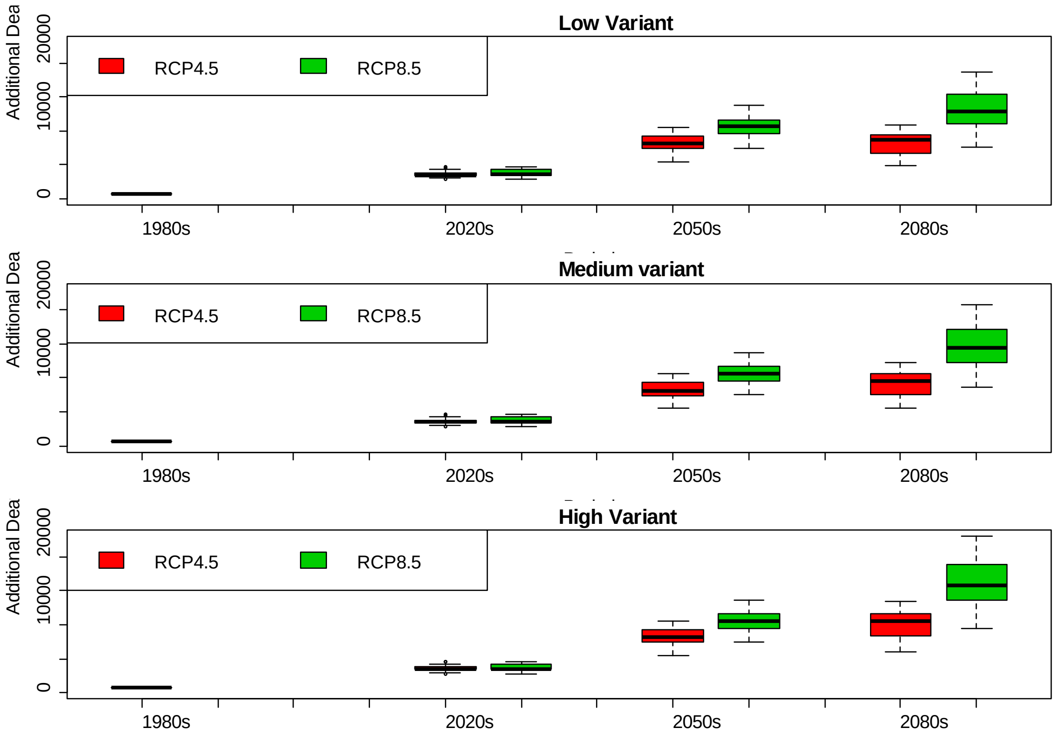

Figure S2. Distribution of heat-related annual deaths in the 1980s, 2020s, 2050s, and 2080s for 31 climate models and the RCP4.5 and RCP8.5 scenarios with population low, median, and high variant scenarios

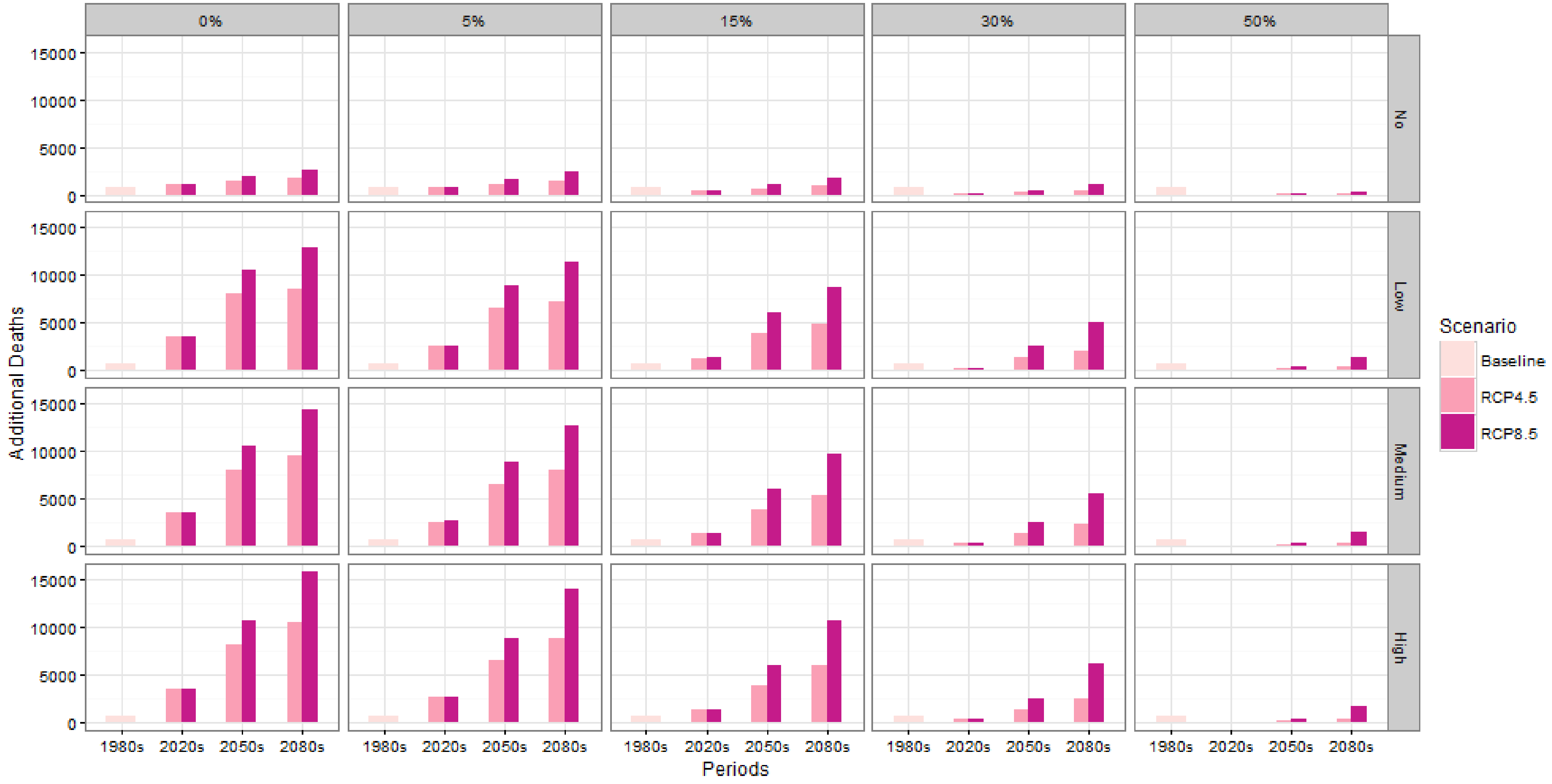

Figure S3. Projection of heat-related deaths (median of 31 models) in the 1980s, 2020s, 2050s and 2080s for different population variant scenarios and the RCP4.5 and RCP8.5 scenarios with different adaptation scenarios.(Rows indicate the population scenarios; Column indicate the adaptation scenarios)

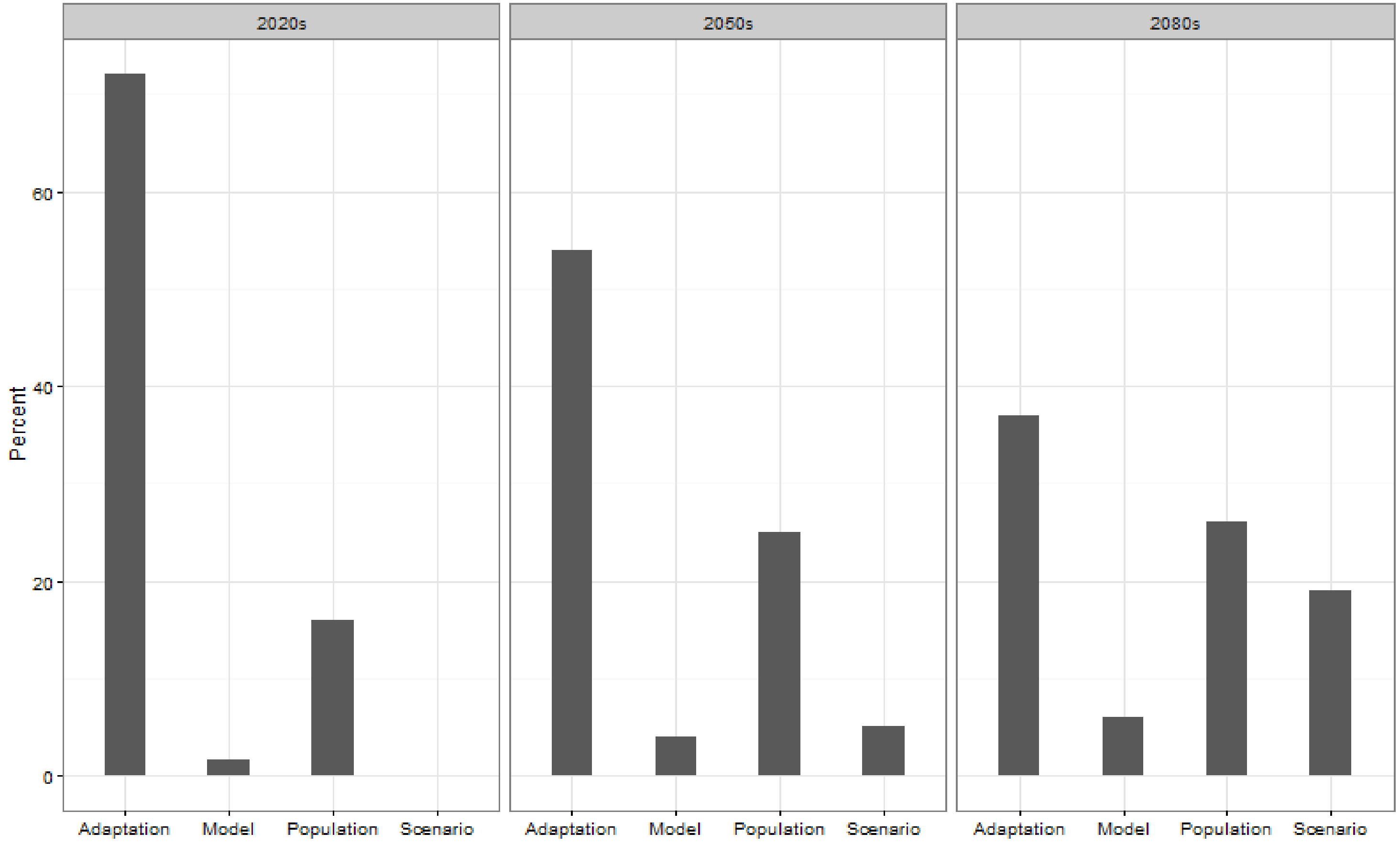

Figure S4. Uncertainty analysis of heat-related mortality projection affected by various factors in different period
